# Counting statistics of photon emissions detected in non-Markovian environment

**DOI:** 10.1038/s41598-017-18980-w

**Published:** 2018-01-11

**Authors:** Luting Xu, Xin-Qi Li

**Affiliations:** 1 Center for Joint Quantum Studies and Department of Physics, Tianjin University, Tianjin, 300072 China; 2 Department of Physics, Beijing Normal University, Beijing, 100875 China

## Abstract

In this work we present a large-deviation analysis for the counting statistics of atomic spontaneous emissions continuously detected in finite-bandwidth non-Markovian environment. We show that the statistics of the spontaneous emissions depends on the time interval (*τ*) of successive detections, which can result in big differences such as dynamical phase transition. This feature excludes the idea of regarding the spontaneous emissions as detection-free *objective* events. Possible experiment is briefly discussed in connection with the state-of-the-art optical cavity set-up.

## Introduction

In quantum theory the problem that the spontaneous emission of photon from an atom is dynamically *objective* or *detector-dependent* is fundamentally important and interesting. At the early stage the quantum ‘jumps’ associated with the photon emissions were conceived of as objective dynamical events^1,2^. However, the later development of quantum mechanics within the framework of quantum wavefunction description implies that the photon emissions can take place only by detection (measurement)^3–6^, in marked contrast to the objective jumps of Bohr and Einstein. Very recently, this problem was revisited by Wiseman *et al*. by showing how different detection schemes can result in different types of jumps^7^, in terms of quantum-mechanically steering the ‘earlier emission event’ by the post-stage detection.

In this work we alternatively make this issue in contact with the counting statistics of the spontaneous emissions. To exclude the picture as objective events, we show that the spontaneous emissions are strongly affected by the time interval (*τ*) in between the moments we check the emissions happened or not. We also show that this demonstration can be fulfilled only by performing the photon detections in a finite-band non-Markovian reservoir^8,9^. Associated with the non-Markovian dynamics of open quantum systems^8,9^, existing stochastic unraveling evolution of the reduced density matrix dynamics cannot be interpreted as measurement-conditioned physical quantum trajectory^10–14^. This is in sharp contrast with the situation of photon-detections in (infinite) wide-band Markovian environment^3–6^, where the *no-effect* of intermediate frequent null-result (no emission registered) measurements makes the ensemble average of the quantum trajectories identical to the usual reduced density matrix. We may explain the *no-effect* issue in more detail by taking the simple example of an atom subject to no more driving but prepared in a superposition of the ground and excited states. Let us imagine to check a photon emitted or not in the environment, over the time duration (0, *t*). In the Markovian case, the many-times of frequent check over (0, *t*) will arrive to the same conclusion as that checking only at the last moment *t*.

**Figure 1:**
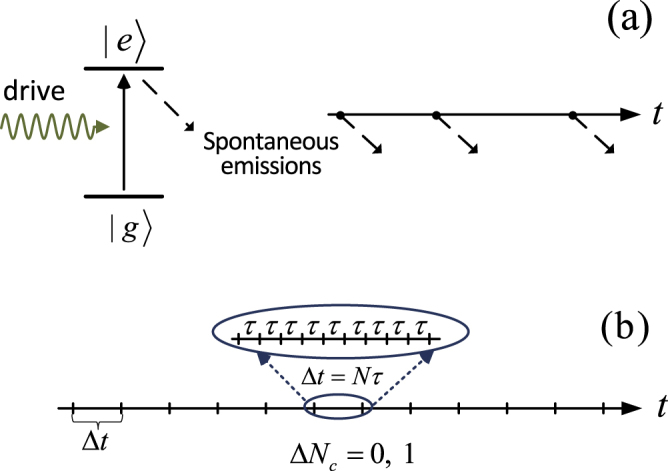
(**a**) Schematic plot for the random spontaneous photon emissions from a driven atom. In the absence of photon detection by introducing outside detector, whether or not the spontaneous emissions take place as *objective* events is of essential importance which actually touches the bottom of quantum theory. (**b**) Successive detection of photons after every short time interval *τ* (to mimic the ‘continuous’ detection by detector with response time *τ*). In order to construct an efficient theory for this type of measurements, the accumulated result over Δ*t* = *Nτ* (determined by Δ*N*_*c*_ = 0 or 1), can be utilized to perform a one-step update for the atom state.

For atoms subject to continuous driving (Rabi oscillation), as schematically shown in Fig. 1, a series of spontaneous photon emissions will take place. We can thus insert the above consideration into the study of counting statistics of the spontaneous emissions, in particular performing a large-deviation (LD) analysis^15–19^. We will show that the results would strongly depend on the time interval *τ* in between the successive photo-detections, which can lead to big differences such as dynamical phase transition. In practice, the time interval *τ* in this theoretical consideration qualitatively corresponds to the response time of photo-detectors. Here the ‘response time’ means the time delay of the output photo-current after the photon to be measured reaches the detector. This is the minimal time interval which allows us to be able to count two successive photons. Note also that, in the standard continuous-photon-detection-based quantum trajectory theory (associated with measurements in Markovian environment), this type of consideration has been involved as well in constructing the continuous measurement theory^3–6^.

## Results

### Model and measurement-results conditioned evolution

Let us consider a driven multi-level atom coupled to the electromagnetic vacuum (environment). For the sake of simplicity, we assume only a single radiative channel, e.g., from |*e*_*j*_〉 to |*g*〉. The total Hamiltonian can be formally expressed as

1 $$H={H}_{S}+\sum _{k}({b}_{k}^{\dagger }{b}_{k}+\mathrm{1/2}){\omega }_{k}+\sum _{k}\,[{V}_{k}{b}_{k}^{\dagger }{\sigma }_{j}^{-}+{\rm{H}}\mathrm{.}{\rm{c}}\mathrm{.}]\,\mathrm{.}$$

Throughout this work we set *ħ* = 1. The optical driving is contained in *H*_*S*_, and the coupling to the environment is via the atomic operators ${\sigma }_{j}^{-}=|g\rangle \langle {e}_{j}|$ and ${\sigma }_{j}^{+}=|{e}_{j}\rangle \langle g|$. *V*_*k*_ is the coupling amplitude of the atom with the environment. The property of the environment (and of the coupling) is largely characterized by the spectral density function (SDF)

2 $$D(\omega )=\sum _{k}|{V}_{k}{|}^{2}\delta (\omega -{\omega }_{k})\to {D}_{0}{{\rm{\Lambda }}}^{2}/[(\omega -{\omega }_{0}{)}^{2}+{{\rm{\Lambda }}}^{2}]\,\mathrm{.}$$

Here we approximated the SDF by a finite-band Lorentzian spectrum with *ω*_0_ the spectral center and Λ the width.

In the absence of detection, the state of the whole system-plus-environment evolves following the Schrödinger equation, under the Hamiltonian of Eq. (1). However, the presence of detection in the reservoir would interrupt this unitary evolution, resulting in the ‘event’ of photon emission (quantum ‘jump’). Conceptually, we assume that one is able to perform this instantaneous detection after every short time interval *τ*. This is equivalent to the *continuous detection* by using real detectors with signal-response time *τ* (see Fig. 1).

To construct an *efficient* theory for the successive photon detections with very short time interval *τ* (to mimic the ‘continuous’ detection), one can utilize the accumulated result over Δ*t* = *Nτ* to perform a one-step update for the atom state, see Fig. 1(b). This longer time duration Δ*t* is determined from the assumption that during Δ*t* there is *at most* one photon registered in the detector^3–6^. Specifically, let us consider the time interval (*t*, *t* + Δ*t*). There will be two possible outcomes: a photon registered in the detector (Δ*N*_*c*_ = 1), or no photon registered (Δ*N*_*c*_ = 0). In the former case, we simply update the atom state by a ‘jump’ action; while for the latter result the atom takes an *effective* smooth (but non-unitary) evolution. Including also the evolution caused by the optical driving, we can update the atom state in a compact way as

3 $$|{\rm{\Psi }}(t+{\rm{\Delta }}t)\rangle ={\mathscr{U}}({\rm{\Delta }}t){{\mathscr{M}}}_{\mathrm{1,0}}({\rm{\Delta }}t)|{\rm{\Psi }}(t)\rangle /||\cdot ||\,,$$

where $||\cdot ||$ denotes the normalization factor. ${\mathscr{U}}$(Δ*t*) describes the unitary evolution owing to the optical driving, while ${\mathscr{M}}$_1,0_(Δ*t*) are the Krause operators in the POVM formalism which read, respectively, ${{\mathscr{M}}}_{1}({\rm{\Delta }}t)={\sigma }_{j}^{-}$ for Δ*N*_*c*_ = 1, and ${{\mathscr{M}}}_{0}({\rm{\Delta }}t)=diag\{\bar{a}({\rm{\Delta }}t),\,\mathrm{1,}\,\cdots ,\,\mathrm{1\}}$ for Δ*N*_*c*_ = 0 and with $\bar{a}({\rm{\Delta }}t)$ given by^20,21^

4 $$\bar{a}({\rm{\Delta }}t)=\exp \{-[\frac{1}{c}-\mathrm{(1}-{e}^{-cx})\frac{1}{{c}^{2}x}]\frac{{\rm{\Gamma }}{\rm{\Delta }}t}{2}\}\,\mathrm{.}$$

In this elegant result, we have introduced the ‘usual’ emission rate Γ = 2*πD*_0_, the frequency off-set parameter via *E* = (*E*_*j*_ − *E*_*g*_) − *ω*_0_ = *d*Λ and *c* = 1 − *id*, and the scaling variable *x* = Λ*τ*. Note also that the above form of ${\mathscr{M}}$_0_(Δ*t*) is associated with expressing the atom state |Ψ(*t*)〉 = *α*_*j*_(*t*)|*e*_*j*_〉 + *β*(*t*)|*g*〉 +… in terms of a column vector [*α*_*j*_(*t*), *β*(*t*), …]^*T*^, which makes well defined the action of ${\mathscr{M}}$_0_(Δ*t*) on the atom state.

From Eq. (4), in the wide-band (Markovian) limit, *x* → ∞ and *c* → 1, one recovers the standard result $\bar{a}({\rm{\Delta }}t)\to {e}^{-{\rm{\Gamma }}{\rm{\Delta }}{\rm{t}}/2}$. On the other hand, in the limit of *x* → 0, one finds from Eq. (4) that $\bar{a}({\rm{\Delta }}t)=1$, so that the atom is frozen in its initial state under frequent measurements, showing the Zeno effect. From Eq. (4), one can also define an *effective* decay rate

5 $${\gamma }_{{\rm{eff}}}(x)={\rm{R}}e\{[1-{(cx)}^{-1}(1-{e}^{-cx})]/c\}\,{\rm{\Gamma }}\mathrm{.}$$

Note that for the wide-band-limit Markovian environment, the result implies *no-effect* of the intermediate *null-result* (no photon detected) interruptions^22^. For finite-bandwidth environment, however, Eq. (5) shows that the decay rate is influenced by the frequent null-result measurements. This *x*- or *τ*-dependence is essentially rooted in the non-Markovian nature of the environment.

### Large-deviation analysis

Below we outline the formalism for analyzing the statistical properties of the dynamical trajectories of the spontaneous emissions^15–19^. Actually, counting statistics of spontaneous emissions is associated with the *ensemble average* over the two possible outcomes leading to Eq. (3). The resultant atom state is thus described by a reduced density matrix which satisfies a master equation^21^. For the purpose of large-deviation analysis, we introduce the *n*-dependent reduced density matrix, *ρ*^(*n*)^(*t*). It describes the atom state conditioned on the total number (*n*) of photons detected over (0, *t*). The equation-of-motion of *ρ*^(*n*)^(*t*) is given by^19^

6 $${\dot{\rho }}^{(n)}=-i[{H}_{S},{\rho }^{(n)}]+{\gamma }_{{\rm{eff}}}(x)({\sigma }_{j}^{-}{\rho }^{(n-\mathrm{1)}}{\sigma }_{j}^{+}-\frac{1}{2}\{{\sigma }_{j}^{+}{\sigma }_{j}^{-},{\rho }^{(n)}\})\mathrm{.}$$

Knowing the *n*-resolved density matrix, we can obtain the LD function *P*(*s*, *t*) via the following transformation

7 $$P(s,t)=\sum _{n}{e}^{-sn}P(n,t)={e}^{- {\mathcal F} (s,t)},$$

where *P*(*n*, *t*) = Tr[*ρ*^(*n*)^(*t*)] and, as to be clear below, ${\mathcal F}$(*s*, *t*) plays the role of *generating* function for the LD analysis. In Eq. (7), the real nature of the transforming factor *e*^−*sn*^ makes the resultant *P*(*s*, *t*) resemble the partition function in statistical mechanics. That is, the trajectories are categorized by a dynamical order parameter “*n*” or its conjugate field “*s*”. In statistical mechanics, the partition function measures the number of microscopic configurations accessible to the system under given conditions. For the spontaneous emissions, if we are interested in the dynamical aspects of the emitted photons, the above insight can lead to an LD analysis in time domain. In particular, it allows to inspect the *rare fluctuations* or *extreme events* by tuning the conjugate field “*s*”.

In practice, instead of solving Eq. (6), we introduce *ρ*(*s*, *t*) = ∑_*n*_*e*^−*sn*^*ρ*^(*n*)^(*t*) to obtain the equation for *ρ*(*s*, *t*)^19^, and are able to straightforwardly compute the LD function *P*(*s*, *t*) by noting that *P*(*s*, *t*) = Tr[*ρ*(*s*, *t*)]. Then, from the generating function ${\mathscr{F}}$(*s*, *t*) = −ln *P*(*s*, *t*), we have

8a $${ {\mathcal F} }_{1}(s,t)\equiv {\partial }_{s} {\mathcal F} (s,t)=\frac{1}{P(s,t)}\sum _{n}n{e}^{-sn}P(n,t)\equiv {\langle n\rangle }_{s},$$

8b $${ {\mathcal F} }_{2}(s,t)\equiv {\partial }_{s}^{2} {\mathcal F} (s,t)=-{\langle {(n-{\bar{n}}_{s})}^{2}\rangle }_{s},$$

and more generally,

8c $${ {\mathcal F} }_{k}(s,t)\equiv {\partial }_{s}^{k} {\mathcal F} (s,t)={(-)}^{(k+\mathrm{1)}}{\langle {(n-{\bar{n}}_{s})}^{k}\rangle }_{s}\mathrm{.}$$

Here, for brevity, we utilized also the notation ${\bar{n}}_{s}$ for 〈*n*〉_*s*_. From these cumulants, we can define a finite-counting-time flux of the emitted photons *I*(*s*, *t*) = ${\mathcal F}$_1_(*s*, *t*)/*t* and the shot noise *S*(*s*, *t*) = 2|${\mathcal F}$_2_(*s*, *t*)|/*t*. Conventionally, one may employ the Fano factor ${\mathcal F}$(*s*, *t*) = ${\mathcal F}$_2_(*s*, *t*)/${\mathcal F}$_1_(*s*, *t*), or the so-called Mandel factor *Q*(*s*, *t*) = −${\mathcal F}$_2_(*s*, *t*)/${\mathcal F}$_1_(*s*, *t*) − 1, to characterize the fluctuation properties.

### Model (I): two-level atom

First we consider a driven two-level atom, described by the Hamiltonian *H*_*S*_ = $\frac{{\rm{\Delta }}}{2}$*σ*_*z*_ + Ω*σ*_*x*_, where *σ*_*z*_ = |*e*〉〈*e*| − |*g*〉〈*g*| and *σ*_*x*_ = |*e*〉〈*g*| + |*g*〉〈*e*|. The damping operator (spontaneous emission from |*e*〉 to |*g*〉) in Eq. (6) is simply given by *σ*^−^ = |*g*〉〈*e*|.

**Figure 2:**
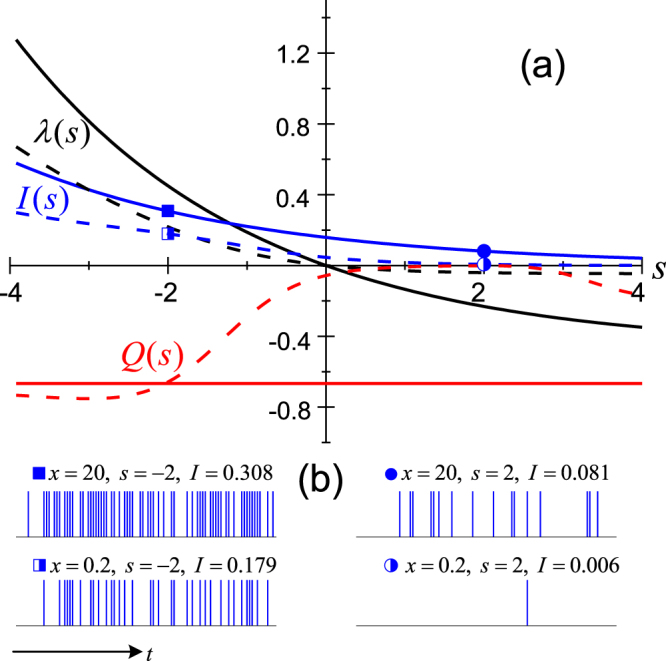
Large-deviation analysis for the spontaneous emission trajectories of a driven two-level atom. (**a**) The characteristic function *λ*(*s*), the flux *I*(*s*) of emitted photons, and the Mandel factor *Q*(*s*) are presented for two sets of trajectories collected by photo-detectors with different response times, which correspond to the “scaling” parameters *x* = 20 (solid lines) and 0.2 (dashed lines). We use a reduced units of system by setting the “natural” spontaneous emission rate Γ = 1, and *γ*_eff_|_*x* = 20_ = 4Ω. (**b**) Representative trajectories from the sub-ensembles as indicated by the specific parameters.

**Figure 3:**
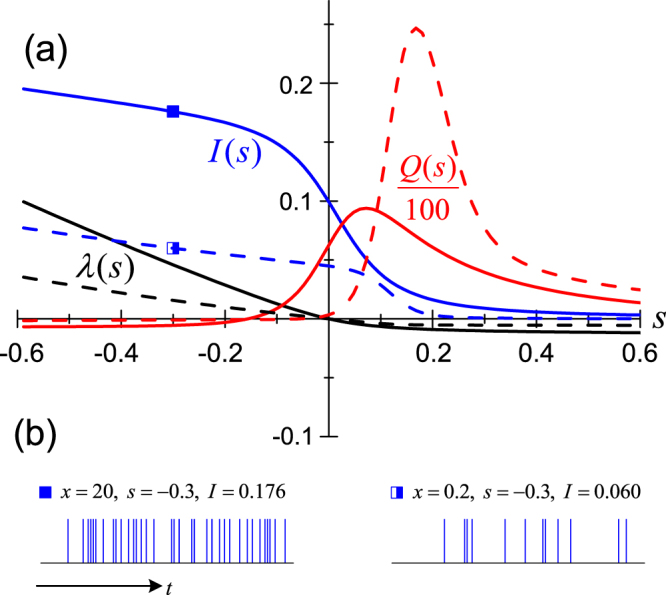
Large-deviation analysis for a driven three-level atom. (**a**) and (**b**): The same plots as described in Fig. 2, and similar reduced units of system adopted by setting Γ = 1, *γ*_eff_|_*x* = 20_ = 4Ω_1_, and Ω_2_ = 0.1Ω_1_.

The simulation result is displayed in Fig. 2. For better understanding to the result presented here, we mention that the LD function around *s* = 0 encodes information of the *typical* trajectories, while away from *s* = 0, on the other hand, it encodes information about the *rare* trajectories via assigning a weight factor *e*^−*sn*^ to select mainly the *active* trajectories (for *s* < 0), or the *inactive* ones (for *s* > 0). In this plot (and in Fig. 3 in the following), we consider a long counting time limit. In this case it can be proved^19^, that the generating function has an asymptotic form ${\mathcal F} (s,t)\simeq t\lambda (s)$, and call *λ*(*s*) the LD *characteristic* function. In Fig. 2, the LD characteristic function *λ*(*s*), the *s*-dependent flux *I*(*s*) of the emitted photons, and the fluctuations–the Mandel factor *Q*(*s*)–are plotted *versus* the conjugated field *s*, as a ‘multi-angle’ characterization for the photon emission trajectories.

The essential point we may stress here is that the statistical properties of the emission trajectories depend on *how often we perform the detections*, in the sense as illustrated in Fig. 1(b). That is, the trajectories *continuously collected* by photo-detector with different response time *τ* may have quite different statistical properties. Note that this is very different from the photon detection in Markovian environment, where the result is *τ* independent. For instance, in Fig. 2 we see that for two different response times, which result in *x* = 20 and 0.2, the photon emission flux *I*(*s*) with *x* = 20 (larger *τ*) is stronger than the result with *x* = 0.2 (smaller *τ*). In particular, the flux *I*(*s* = 0) of the typical trajectories in the case *x* = 0.2 almost vanishes, which actually indicates the Zeno effect since the very frequent detections prevent the spontaneous emission.

More interesting is the behavior of the fluctuations of the *s*-dependent trajectories. For *x* = 20 (we have purposely chosen this parameter), we see that the Mandel factor *Q*(*s*) is an *s*-independent constant, which means a homogeneous fluctuation property. In other words, all the sub-ensemble trajectories collected with *x* = 20 have the same fluctuations. However, if we alter the detection time interval (*x* = 0.2), the fluctuations of the sub-ensemble trajectories are no longer homogeneous, but *s*-dependent as shown in Fig. 2 by the *Q*(*s*) curve.

### Model (II): three-level atom

The second example is the LD analysis for the spontaneous emissions from a three-level atom. The atom is driven by two resonant lasers with Rabi couplings Ω_1_ and Ω_2_, describe by the Hamiltonian *H*_*S*_ = ∑_*j* = 1,2_($\frac{{{\rm{\Delta }}}_{{\rm{j}}}}{2}$*σ*_*jz*_ + Ω_*j*_*σ*_*jx*_), where *σ*_*jz*_ = |*e*_*j*_〉〈*e*_*j*_| − |*g*〉〈*g*| and *σ*_*jx*_ = |*e*_*j*_〉〈*g*| + |*g*〉〈*e*_*j*_|. We assume only one spontaneous emission channel, i.e., from |*e*_1_〉 to |*g*〉. So the damping operator in Eq. (6) reads ${\sigma }_{1}^{-}=|g\rangle \langle {e}_{1}|$.

The result of LD analysis for this driven three-level atom is shown in Fig. 3. For *s* < 0 the active phase corresponds to plentiful photons emitted and most occupation of the state |*e*_1_〉, while for *s* > 0 the inactive phase means that the number of emitted photons is small and the occupation is largely in the state |*e*_2_〉. (Note that the spontaneous emission from |*e*_2_〉 to |*g*〉 is forbidden as we have assumed). Compared to the two-level atom studied above, in the active side (*s* < 0), the behaviors are similar. However, in the inactive side (*s* > 0), the difference is remarkable. The most prominent feature is the appearance of a ‘crossover’ behavior between two distinct dynamical phases. This is most clearly revealed by the Mandel factor *Q*(*s*), where the ‘sharp peak’ indicates the ‘crossover’ between two distinct phases (on the two sides of the peak), as we vary the LD parameter (*s*) through the peak region.

Actually, the crossover behavior is something of a *smoothed first-order phase transition*. We may understand this interpretation in more detail as follows. The peak of *Q*(*s*) in Fig. 3 simply means strong fluctuations of the sub-ensemble trajectories, which are a consequence of fact that the sub-ensemble is a *mixture* of two types of trajectories, i.e., the relatively active and inactive ones (on the two sides of the ‘peak’). In alternative words, the sub-ensemble is a mixture of two distinct dynamical phases. The active phase is that on the left side of the peak and the inactive phase is the one on the right side. We know that *coexistence of two distinct phases* is the physical reason of strong fluctuations, which resembles actually what happens at the critical point (critical temperature) of the first-order (thermal dynamic) phase transition. Since the strong fluctuations appear in the proximity around the peak (but not precisely at a unique critical point of *s*), we may say that, when crossing the round peak, the system experiences a ‘smoothed’ first-order dynamical phase transition, more specifically, a transition from photon-emission-active phase to inactive phase.

The crossover behavior (of suffering a dynamical phase transition) is a consequence of the interplay between the two channels of driving, i.e., |*g*〉 ⇔ |*e*_1_〉 and |*g*〉 ⇔ |*e*_2_〉, and that only on |*e*_1_〉 the photon emission is allowed while on |*e*_2_〉 it is forbidden. Similar statistics behavior of dynamical trajectories was found also in the transport through a parallel double-dot system with Coulomb blockade^19^ where the interplay of the Coulomb blockade and quantum interference induces two effective transport channels, one is slow and another fast.

Again, in Fig. 3, we plot the results from two sets of trajectories with different photon-detection time intervals, i.e., *x* = 20 and 0.2. We find that the crossover behavior for the *x* = 0.2 trajectories is more striking. In the inactive (*s* > 0) regime, the characteristic function *λ*(*s*) is more flat and the flux *I*(*s*) of the emitted photons vanishes more rapidly, meanwhile the *Q*(*s*) peak is much higher and shifts towards larger conjugate field *s* (more inactive subensemble trajectories). *We stress that this τ-dependent feature is unique only for continuous detection of photons in a non-Markovian environment, which does not happen for detection in Markovian environment*.

## Discussion

We have presented a counting statistics study at the level of large-deviation analysis, for atomic spontaneous emissions continuously detected in a non-Markovian environment with finite-bandwidth (Λ). We showed that the statistics behaviors can be strongly influenced by the response time (*τ*) of the detector, via the elegant scaling variable *x* = Λ*τ*. The feature that the trajectories of the spontaneous emissions depend on how often we perform the detections definitely excludes the idea of regarding the spontaneous emissions as detection-free *objective* events. This is because the detection interval *τ* is small enough compared to the average time between the successive spontaneous emissions, thus there are no photons missed in the counting collection. If the spontaneous emissions were *objective*, the statistical properties must be independent of *τ*. Via the scaling variable (*x* = Λ*τ*) analysis, we also showed that it is impossible to demonstrate in Markovian environment the effect of the detection time *τ* on the counting statistics.

In this work we have restricted our analysis to Lorentzian spectrum. However, the above conclusion is valid to arbitrary SDF of non-Markovian environment such as the Ohmic, sub-Ohmic, and super-Ohmic baths. Actually, we have recently generalized the measurement theory and the associated quantum trajectory approach to environment beyond the Lorentzian spectrum^23^. For arbitrary SDF, we proved in general the existence of scaling property. Despite that analytical result is not available in general case, we developed reliable numerical scheme to simulate the quantum trajectories.

As possible implementation in experiment, one may consider to put the atom in the state-of-the-art optical cavity. The cavity mode coupled to outside (Markovian) world is a good finite-bandwidth non-Markovian environment, and is well described by the Lorentzian spectral density function. One can then perform detection for the photons leaked from the cavity. In this set-up, the bandwidth Λ can be modulated by the leaky rate of the cavity photon, to alter the scaling variable *x* = Λ*τ*. This is equivalent to altering the detection time *τ*.

We notice that the spontaneous emissions (resonance fluorescence) from driven artificial atom in superconducting circuit-QED system have been detected in recent experiments^24–29^. However, owing to that direct detection of single photons at microwave frequencies is not yet available at present stage, the quadratures of the microwave-photon-field are measured in these experiments, based on the homodyne or heterodyne detections. Statistics analysis of this type of measurement records is an interesting open question worth future exploration, especially from the perspective of measurement in non-Markovian environment as considered in present work. As a final remark, we mention also the recent interests in the *most-likely-paths* (MLP) among the huge number of stochastic quantum trajectories under continuous monitoring^29–31^. In this context, it would be of interest to study the statistics of the sub-ensemble of ‘rare events’ (rare paths), in similar sense of the LD studies.
